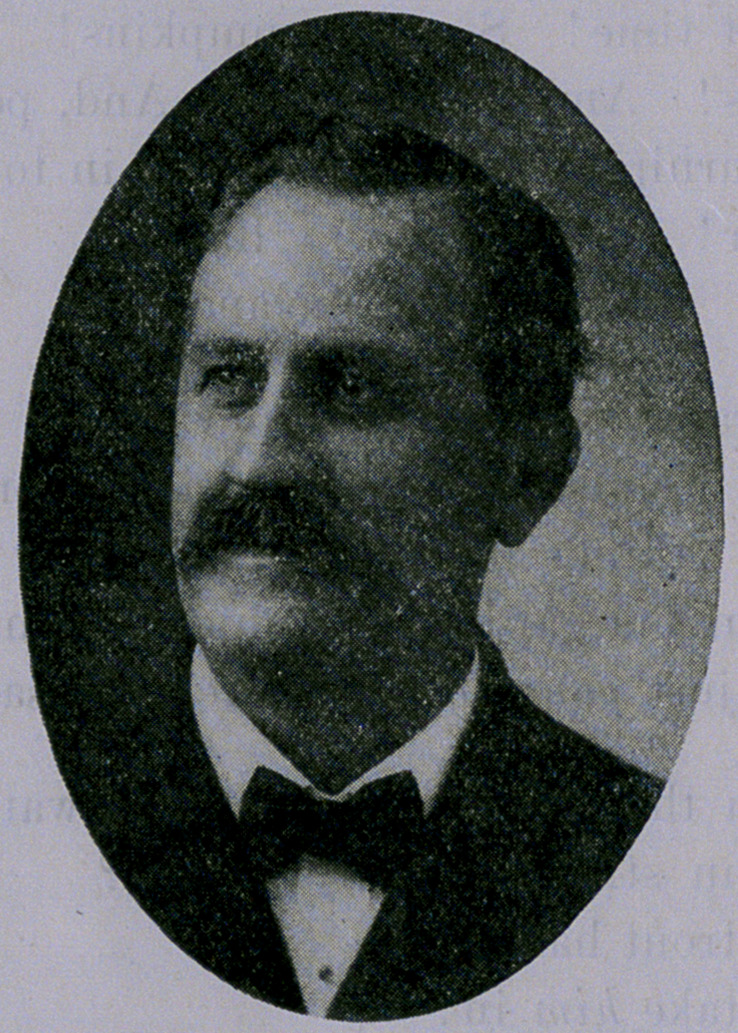# Death of Dr. Fry

**Published:** 1911-10

**Authors:** 


DEATH OF DR. FRY.—Our esteemed friend, Dr. J. M. Fry, died at his home in Wills Point, Texas, Sunday, September 10, 1911, aged 61. We are indebted to the Wills Point *Chronicle* for the cut and data.

Dr. Fry was born in Tennessee, October, 1850. He was a small boy but he joined the Confederate army (Eighth South Carolina Infantry); later he was a scout under Morgan. At the surrender he was a prisoner in Knoxville and was paroled April, 1865. His Confederate record was his most cherished memory, and he never tired talking of the stirring incidents in his career. He took an active interest in the U. C. V. and was Vice-President (Third) of the Association of Army and Navy Medical Officers. He was a graduate of Kentucky School of Medicine, 1876; an active member of State, County and Tri-State Medical Societies. He was recognized as a physician of great skill, and his admirable social qualities endeared him to all who knew him. His son, Dr. H. T. Fry, a daughter, two brothers and three sisters survive him. Peace to his ashes!

## Figures and Tables

**Figure f1:**